# The limits of defaults: why french fries trump apple slices

**DOI:** 10.1186/s13104-016-2061-z

**Published:** 2016-05-06

**Authors:** Brian Wansink, David R. Just

**Affiliations:** Charles H. Dyson School of Applied Economics and Management at Cornell University, 210C Warren Hall, Ithaca, NY 14853 USA

**Keywords:** School age children, Food choices, Behavior, Obesity, Fast food, Defaults

## Abstract

**Background:**

Healthy default food choices have been suggested as a way to encourage better nutrition without restricting choice. Will they work with children and their favorite foods?

**Methods:**

A group of children, 6–8 years old, were treated to lunch at fast food restaurant on 2 days 2 weeks apart. On both days the children were served chicken nuggets and a drink. On the first day, half were given French fries unless they asked for apple slices and the other half were given apples unless they asked for fries. The order switched on the second day.

**Results:**

When the default changed from fries to apples, 86.7 % opted out of the default to order fries.

**Conclusion:**

Defaults may be ineffective when children have a strong preference for the less healthy option. Allowing children to take both sides may lead to healthier consumption than constructing an artificial default choice.

## Background

Childhood obesity is partially driven by the unhealthy choices kids make in restaurants, school lunchrooms, and fast food venues. Behavioral scientists have widely suggested the simple solution is to make the healthy choice the default choice [1]. For instance, fast food restaurants could offer all children’s meals with healthy side options so that if a child wants the less healthy option, they have to ask. The hesitancy to “opt-out” of a default has been shown to sizably increase the percentage of adults who tacitly agree to organ donation and payroll deductions for retirement [2, 3]. Default choices are effective with adults in contexts where they are indifferent or have no strong preference, but will it work with children and their favored foods?

## Methods

All 15 of the 6–8 year old, multi-ethnic children (6 male) attending an upstate New York summer camp in July 2011 participated in a Cornell IRB-approved study. All children at the camp came from a housing development in which tenants received significant government assistance with their rent, and were thus likely from households with relatively low incomes. Physical consent forms were given to parents along with verbal explanations the week prior to the study. Children were also asked if they were willing to participate at that time. Children were all given children’s meals from a popular national fast food chain on two separate days 1 week apart. On both days the children were given chicken nuggets and a drink. In the first week, half were given French fries unless they asked for apple slices and the other half were given apple slices unless they asked for French fries. This order was switched in the second week so every child was given both apple slices and French fries as a default during one of the 2 weeks.

The choice of a side took place in the following way. In both conditions, subjects received a packet of the default side, and were told not to open (in the case of apples) or eat the packet, but count how many pieces were inside. Once they had the packet in their possession for about 10 min, they were asked to go up to a researcher one by one, and tell how many apple slices were inside. Similar methods have been used in prior studies to establish an endowment effect which some theorize to be the power of defaults in choice [4–6]. The researcher then offered them a choice to swap apple slices for French fries. Their choices were noted, and plate waste was measured.

### Statistical analysis

We assume that the selection of French fries or apple slices is distributed Bernoulli, with a fixed probability θ that each child will choose the default. Thus, the sum of those choosing the default option is binomially distributed with parameters *n* = 15 and θ, where θ is the parameter of interest to be estimated. Comparing any two samples, we will be able to reject that both have the same probability of selecting the default at the *p* = 0.05 level if we observe a difference of 6 choosing the default option, and at the *p* = 0.10 level if we observe a difference of 5, which would correspond to 1/3 of the children changing their choice between treatments. Given the large effects claimed in the literature [2, 3] on defaults—on the order of 60 %—we find that this sample size is large enough to determine if the effects are of similar magnitude.

## Results and discussion

When French fries were the default option, only one of fifteen children chose to switch from French fries to apple slices (6.7 %). In contrast, when apple slices were the default, all but two children chose to switch from apple slices to French fries (86.7 %). The difference in the percent switching from the default is fully 80.0 % (p < 0.001). Thus children responded very differently to the healthy default than to the unhealthy default.

All of the selected French fries were eaten on both days, and nearly all of the selected apple slices were consumed. In total, the percentage eating apple slices when French fries were the default was 6.7 %, while 13.3 % consumed apple slices when apple slices were the default. Statistically this difference was insignificant (p = 0.58). The default had no discernible impact on consumption of apples or French fries, though there was a significant difference in the decision to switch from the default.

In contrast to non-food-related research on defaults [3], these results suggest that defaults may not be effective with some of the most common choices. Admittedly, our pilot experiment uses a very small sample and the results must be treated with care. While our sample is large enough to detect effects of around 1/3 of the sample, a larger sample would be required to detect smaller impacts on choice. We used a procedure that emphasized the endowment effect in our default. An alternative view of defaults supposes their power comes from an individual’s lack of deliberate decision-making. Our procedure could have undermined such an effect by giving children significant time to consider their choice. Moreover, our procedure is not particularly practical and could not easily be implemented in a real world setting. In a quick service restaurant, it may not be fully obvious that other options are available, which could make defaults more powerful.

## Conclusion

Default options in food choices may not be a realistic solution for encouraging better food choices [7]—particularly in a fast-food restaurant. Our design gave the 6–8 year-old children every possibility to feel endowed with the apple slices, yet it still made no difference in what they chose and consumed.

The use of defaults may also dangerously lead to binary, either/or choices—a child either eats the healthy food or they eat the unhealthy food. One solution would be to provide both options. Perhaps offer them a reduced amount of French fries and a serving of apple slices [8]. While this solution is admittedly less healthy than eating only the apple slices, the latter outcome may be unrealistic.
